# On the reporting and analysis of a cancer evolutionary adaptive dosing trial

**DOI:** 10.1038/s41467-020-20174-4

**Published:** 2021-01-12

**Authors:** Hitesh B. Mistry

**Affiliations:** Division of Pharmacy, University of Manchester, Manchester, UK

**Keywords:** Cancer, Prostate cancer, Evolution, Applied mathematics

**Arising from** J. Zhang et al. *Nature Communications* 10.1038/s41467-017-01968-5 (2017)

There is growing interest in applying mathematical models/methods from ecology to cancer, such as adapting the dose based on the level of tumor burden, i.e., evolutionary-based adaptive dosing. The first trial of such an approach based on mathematical modeling reported that adaptive dosing was superior to continuous dosing based on a single cohort of 11 patients. The paper though lacked details on patient characteristics, acknowledgement of known confounders, and improper comparisons to historical controls. Thus, the evidence that adaptive dosing is superior to continuous dosing still does not exist, and so patients should remain on continuous dosing until a well-conducted randomized-control trial is performed.

The study by Zhang et al.^1^ reports on the initial results of the first cancer clinical trial employing an evolutionary-based adaptive dosing algorithm within the clinic. The study was conducted using the drug Abiraterone within the metastatic castration-resistant prostate cancer (mCRPC) setting. The initial results were based on 11 patients who had their treatment stopped and started based on a simple rule relating to prostate-specific antigen (PSA) falls and rises as follows. Patients had to have experienced a PSA fall to be eligible for the study, once a >50% fall was seen, patients had their treatment stopped and restarted once their PSA levels reached pretreatment levels. The final conclusion from their initial analysis was that “The outcomes show significant improvement over published studies and a contemporaneous population.” This conclusion was based on comparing their results against a 16-patient contemporaneous cohort and a clinical trial cohort of 546 patients. This short paper will highlight key issues with these comparisons that can be used to dispute the key conclusion.

The authors compare the radiological progression times of their 11 patients on an adaptive dosing schedule with 16 patients who have received continuous therapy. However, the authors do not provide any information on key prognostic factors of Abiraterone for either their 11 patients or the 16 patients they compare to. In fact, there is no information on the patient characteristics of the 16- contemporaneous cohort. There are numerous prognostic factors for Abiraterone, lactate dehydrogenase, alkaline phosphatase, and hemoglobin to name a few, which were released in the statistical review held at the FDA. Therefore, without information on these prognostic factors, the authors cannot exclude that the difference in progression times was not attributable to differences in prognostic factors between the two cohorts.

The second comparison made by the authors is with the results of a Phase III clinical trial^2^. In the authors’ study, patients were only eligible for the study if they experienced a PSA fall >50%. However, this was not the case in the trial they compare their results to where patients recruited had not yet had any dose of Abiraterone. Therefore, the comparison made by the authors is incorrect as the two cohorts of patients are not necessarily the same.

In summary, the points raised within this short paper highlight that the evidence for adaptive therapy being superior to continuous therapy still does not exist. By highlighting the flaws in reporting and comparisons, it is hoped that the community raises its standards for future trials of this type, especially when making comparisons with historical data.

## Data Availability

There is no new data presented in this paper. Please see Zhang et al.^1^ for original data.
